# Supplementary material for: Nature Photographs as Complementary Care in Chemotherapy: A Randomized Clinical Trial
Source: Int J Environ Res Public Health. 2023 Aug 10;20(16):6555. doi: 10.3390/ijerph20166555 (PMC10454289; doi:10.3390/ijerph20166555)
Supplement: Supplementary file 1 [file ijerph-20-06555-s001.zip › S4_Booklet_Miscellany_Images.pdf]

e-NATURE VIDEO: MISC

CATEGORIES: WATER, TREES, PALE BIRDS, COLORFUL BIRDS, SKY, FLOWERS, INSECTS AND LANDSCAPES

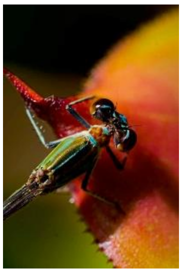

Valence (pleasure) rating: 5.3
Arousal/relaxation rating: 6.1

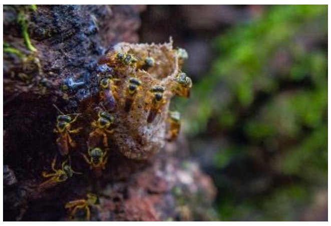

Valence (pleasure) rating: 6.1
Arousal/relaxation rating: 5.7

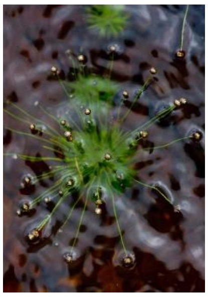

Valence (pleasure) rating: 5.9
Arousal/relaxation rating: 5.2

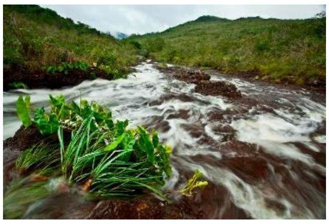

Valence (pleasure) rating: 6.5
Arousal/relaxation rating: 5.4

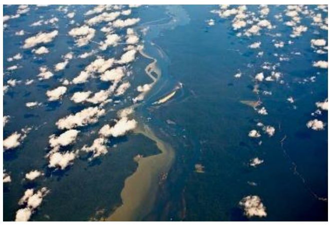

Valence (pleasure) rating: 6.3
Arousal/relaxation rating: 5.1

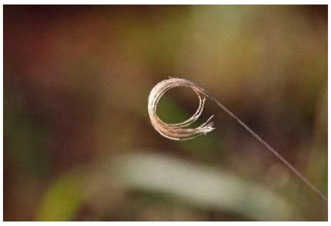

Valence (pleasure) rating: 5.7
Arousal/relaxation rating: 4.7

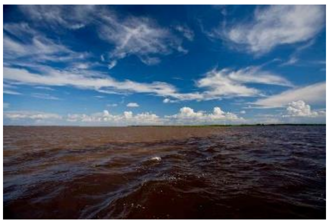

Valence (pleasure) rating: 5.5
Arousal/relaxation rating: 6.1

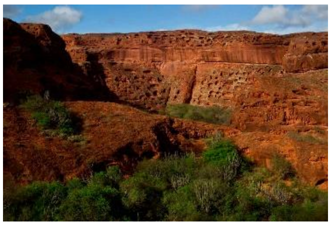

Valence (pleasure) rating: 5.8
Arousal/relaxation rating: 5.3

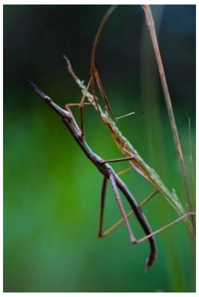

Valence (pleasure) rating: 6.2
Arousal/relaxation rating: 5.3

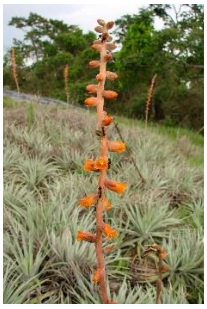

Valence (pleasure) rating: 6.1
Arousal/relaxation rating: 5.0

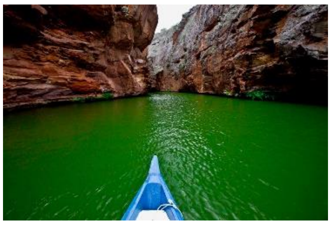

Valence (pleasure) rating: 6.9
Arousal/relaxation rating: 5.1

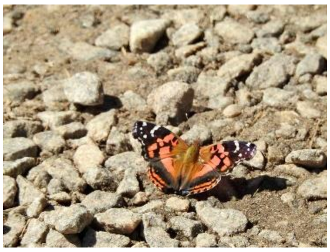

Valence (pleasure) rating: 5.6
Arousal/relaxation rating: 4.6

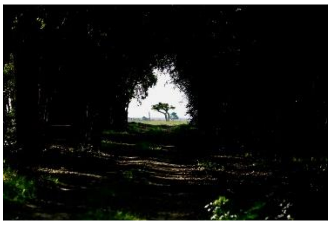

Valence (pleasure) rating: 6.3
Arousal/relaxation rating: 4.7

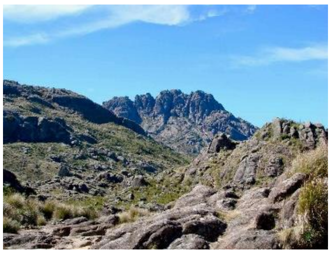

Valence (pleasure) rating: 6.6
Arousal/relaxation rating: 4.7

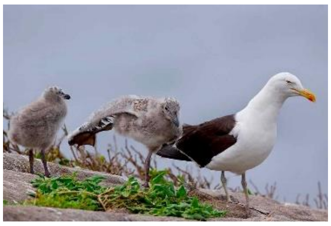

Valence (pleasure) rating: 6.4
Arousal/relaxation rating: 4.5

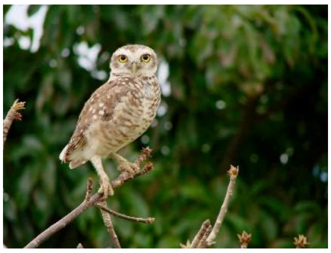

Valence (pleasure) rating: 7.1
Arousal/relaxation rating: 4.9

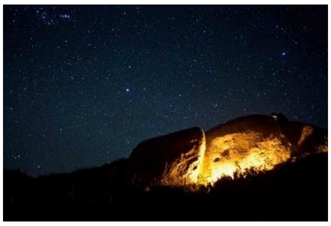

Valence (pleasure) rating: 6.6
Arousal/relaxation rating: 4.3

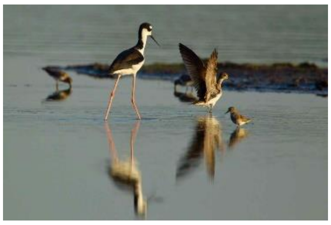

Valence (pleasure) rating: 6.6
Arousal/relaxation rating: 4.3

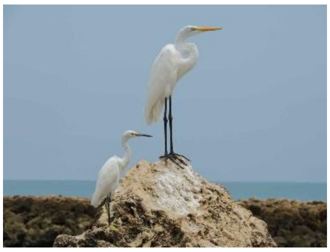

Valence (pleasure) rating: 6.7
Arousal/relaxation rating: 4.0

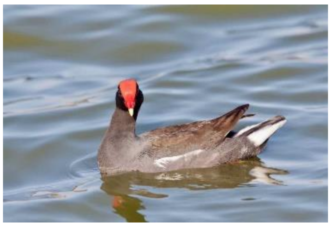

Valence (pleasure) rating: 6.8
Arousal/relaxation rating: 3.9

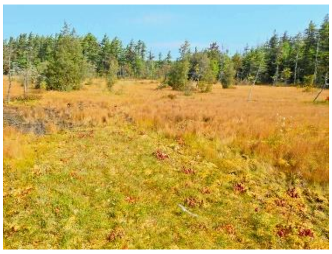

Valence (pleasure) rating: 6.2
Arousal/relaxation rating: 4.6

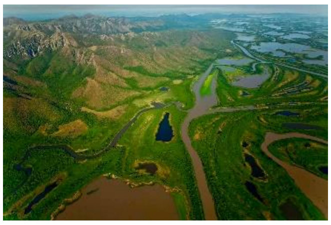

Valence (pleasure) rating: 6.5
Arousal/relaxation rating: 4.8

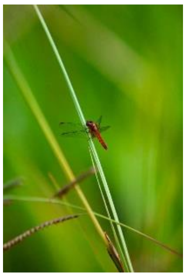

Valence (pleasure) rating: 6.5
Arousal/relaxation rating: 4.6

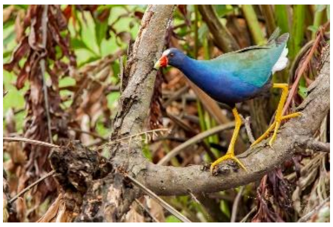

Valence (pleasure) rating: 6.8
Arousal/relaxation rating: 4.6

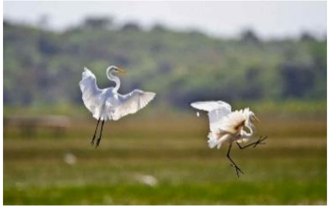

Valence (pleasure) rating: 7.0
Arousal/relaxation rating: 4.5

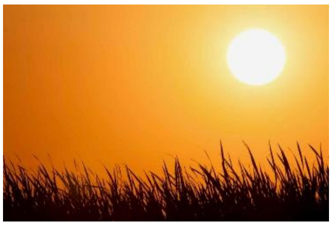

Valence (pleasure) rating: 6.9
Arousal/relaxation rating: 4.3

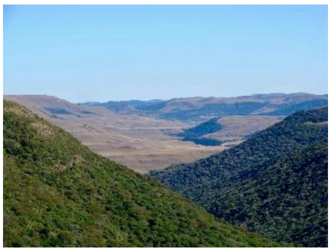

Valence (pleasure) rating: 6.7
Arousal/relaxation rating: 4.0

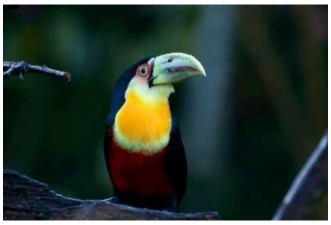

Valence (pleasure) rating: 7.1
Arousal/relaxation rating: 4.3

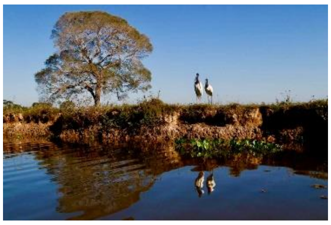

Valence (pleasure) rating: 6.8
Arousal/relaxation rating: 3.9

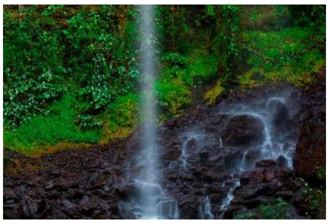

Valence (pleasure) rating: 7.1
Arousal/relaxation rating: 4.2

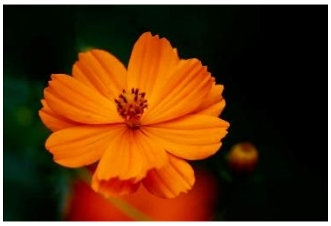

Valence (pleasure) rating: 7.1
Arousal/relaxation rating: 4.2

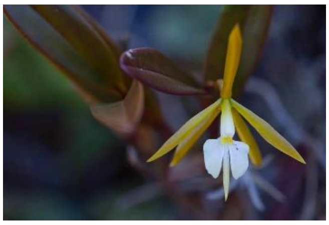

Valence (pleasure) rating: 6.7
Arousal/relaxation rating: 3.8

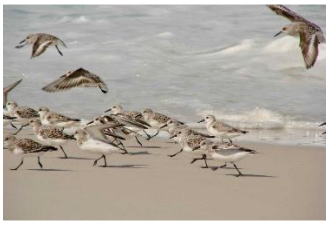

Valence (pleasure) rating: 7.4
Arousal/relaxation rating: 4.4

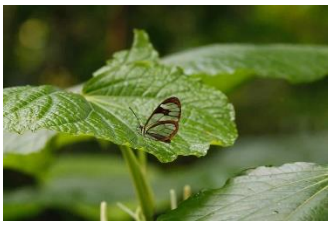

Valence (pleasure) rating: 6.8
Arousal/relaxation rating: 3.7

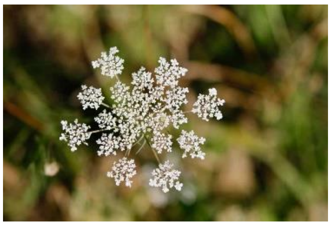

Valence (pleasure) rating: 7.0
Arousal/relaxation rating: 3.8

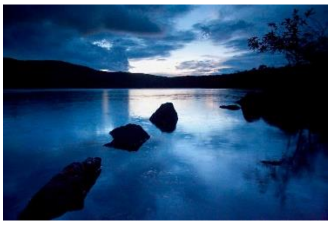

Valence (pleasure) rating: 6.5
Arousal/relaxation rating: 3.3

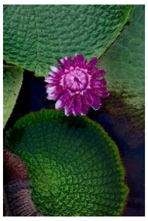

Valence (pleasure) rating: 7.1
Arousal/relaxation rating: 4.2

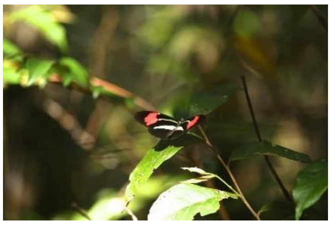

Valence (pleasure) rating: 6.8
Arousal/relaxation rating: 3.9

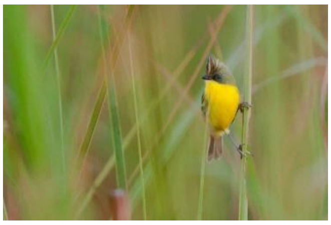

Valence (pleasure) rating: 7.0
Arousal/relaxation rating: 4.1

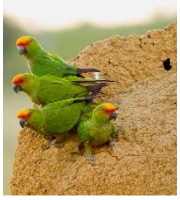

Valence (pleasure) rating: 7.5
Arousal/relaxation rating: 4.5

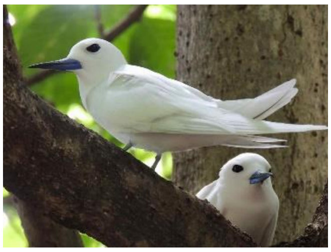

Valence (pleasure) rating: 7.2
Arousal/relaxation rating: 4.2

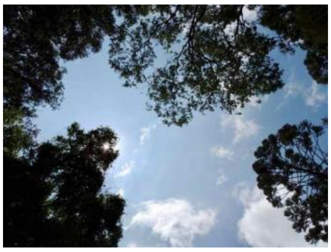

Valence (pleasure) rating: 7.0
Arousal/relaxation rating: 3.9

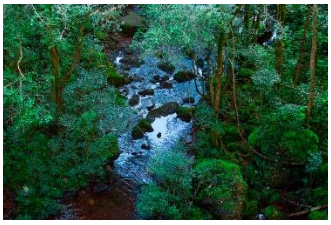

Valence (pleasure) rating: 7.1
Arousal/relaxation rating: 3.9

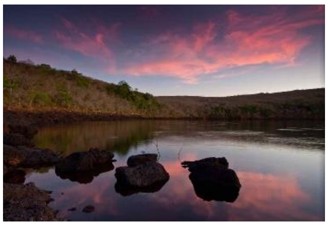

Valence (pleasure) rating: 6.7
Arousal/relaxation rating: 3.5

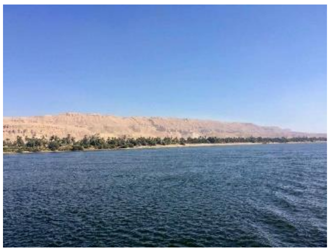

Valence (pleasure) rating: 6.7
Arousal/relaxation rating: 3.4

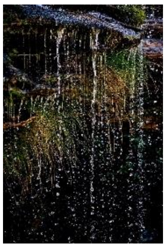

Valence (pleasure) rating: 7.0
Arousal/relaxation rating: 3.7

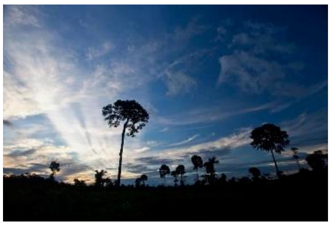

Valence (pleasure) rating: 7.3
Arousal/relaxation rating: 3.6

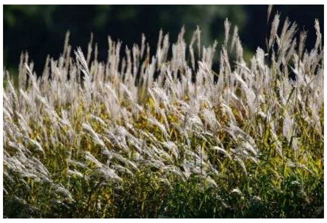

Valence (pleasure) rating: 6.8
Arousal/relaxation rating: 3.5

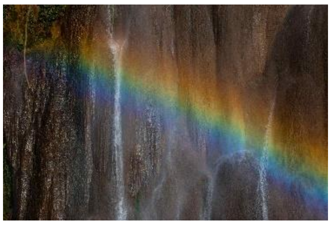

Valence (pleasure) rating: 7.1
Arousal/relaxation rating: 3.7

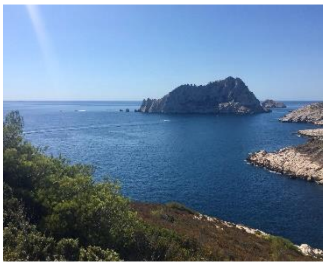

Valence (pleasure) rating: 7.3
Arousal/relaxation rating: 3.8

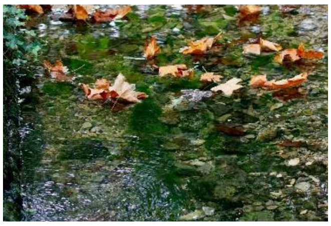

Valence (pleasure) rating: 6.9
Arousal/relaxation rating: 3.4

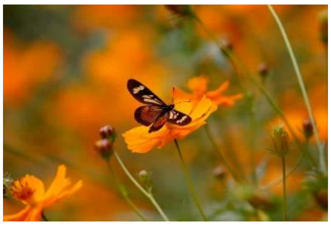

Valence (pleasure) rating: 7.3
Arousal/relaxation rating: 3.7

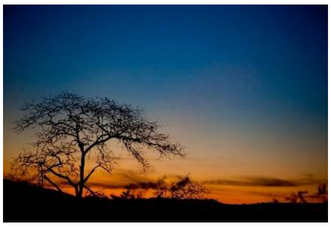

Valence (pleasure) rating: 6.7
Arousal/relaxation rating: 3.0

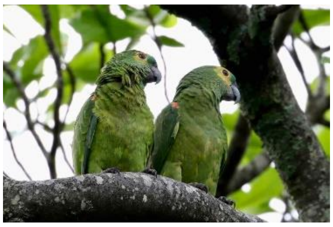

Valence (pleasure) rating: 7.3
Arousal/relaxation rating: 3.4

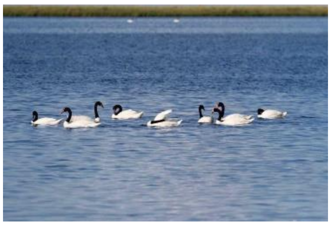

Valence (pleasure) rating: 7.2
Arousal/relaxation rating: 3.4

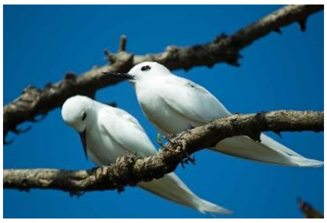

Valence (pleasure) rating: 7.1
Arousal/relaxation rating: 3.2

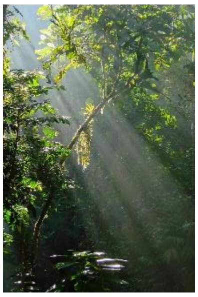

Valence (pleasure) rating: 7.2
Arousal/relaxation rating: 3.3

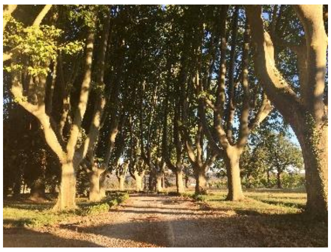

Valence (pleasure) rating: 7.5
Arousal/relaxation rating: 3.1
